# Mitral valve leaflet blood cyst treated with minimally invasive approach: a case report and review of literature

**DOI:** 10.1186/s13019-024-02493-5

**Published:** 2024-01-28

**Authors:** Matiullah Masroor, Ting Xie, Dayan Yang, Shengxiong Lin, Nianguo Dong, Fujin Liu, Long Wu

**Affiliations:** 1 Department of Cardiovascular Surgery, Union Hospital, Tongji Medical College, Huazhong University of Science and Technology, Wuhan, 430022 China; 2 Department of Cardiothoracic and Vascular Surgery, Amiri Medical Complex, Qargha Road, Afshar, Kabul, Afghanistan; 3 Department of Cardiac Surgery, Hainan General Hospital, Hainan Affiliated Hospital of Hainan Medical University, Haikou, 570311 China; 4 Department of Ultrasound Medicine, Hainan General Hospital, Hainan Affiliated Hospital of Hainan Medical University, Haikou, 570311 China; 5 Department of Pathology, Hainan General Hospital, Hainan Affiliated Hospital of Hainan Medical University, Haikou, 570311 China

**Keywords:** Benign heart tumors, Cardiac blood cyst, Mitral valve, Minimally invasive surgery

## Abstract

**Introduction:**

Cardiac blood cyst is a very rare benign tumor of the heart in adults. Though it is very common in the first half year of life, it regresses with time and its occurrence is very rare in children older than six months and in adults. Until now less than 100 valvular blood cyst cases have been reported in adults.

**Case presentation:**

We present a case of a 66-year-old male who presented to us with exertional chest tightness, shortness of breath, and right leg weakness for two weeks. He was diagnosed with a cardiac mass two months ago in another hospital. The physical examination was unremarkable. Abdominal ultrasound showed a cyst in the liver and left kidney. Echocardiography showed a mass-occupying lesion of a cystic nature in the mitral valve with moderate mitral regurgitation. Based on echocardiography findings and computed tomography report, the preliminary diagnosis of mitral valve cystic tumor was made. The patient underwent minimally invasive resection of the cyst. The posterior mitral cusp was repaired and a mitral annuloplasty ring was placed. The postoperative recovery was uneventful. The histopathology report confirmed the diagnosis of a cardiac blood cyst. The patient was followed up for six months without any complications. This case is presented to enrich the medical literature on the cardiac blood cyst.

**Conclusion:**

Although a cardiac blood cyst is a rare entity in adults, it still should be considered in the differential diagnosis of cardiac tumors. Because the natural history and hemodynamic effects are very diverse, large symptomatic cardiac blood cysts, especially in the left heart should be resected to avoid complications.

## Introduction

Primary cardiac tumors are very rare with an incidence of 0.02% [1, 2]. About 74–95% of primary heart tumors are benign [1, 3]. Cardiac myxoma is the most common of the benign primary heart tumors accounting for half of all primary heart tumors [1, 2, 4, 5]. Cardiac blood cyst is a rare kind of benign tumor with limited cases reported in adults. It was first described by Elsasser in 1844 [6]. Its incidence in fetuses, neonates, and infants range from 25% to almost 100% on autopsy [6]. It usually occurs on the atrioventricular valves of the heart but can occur anywhere [7–9]. Because the tumor is a congenital malformation, it is therefore common in infants under the age of two months but regresses automatically in the first half year of life [10], though, it is very rare in children above six months and in adults. Most of the patients are asymptomatic but they can present with a variety of symptoms depending on the size and location of the tumor such as dyspnea, syncope, or signs and symptoms of embolism which warrants treatment. Echocardiography is the initial investigation of choice for cardiac blood cyst like all other heart tumors [5]. Most of the symptomatic cases are treated surgically because of the risk of developing complications [1, 7, 11]. Here we present a case of mitral valve blood cyst in a 66-year-old male patient who was treated with minimally invasive surgery to enrich the literature.

## Case presentation

**Fig. 1:**
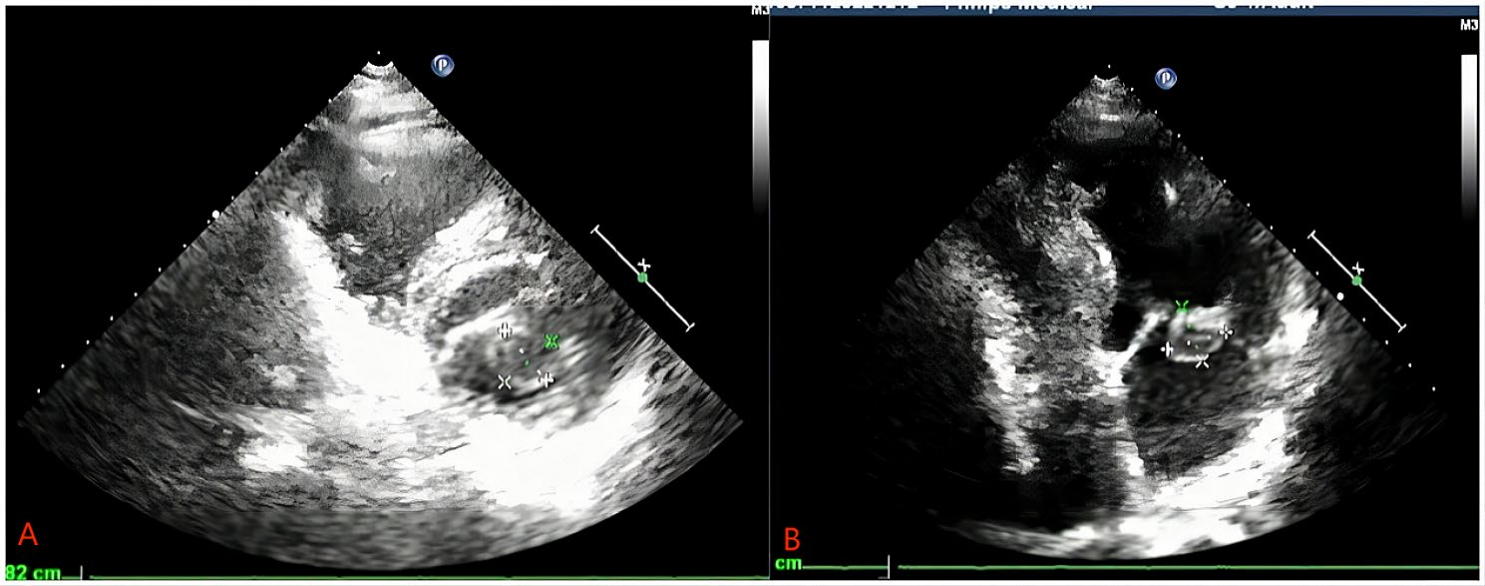
Echocardiography shows a cystic mass attached to the posterior leaflet of the mitral valve containing liquid (A,B)

**Fig. 2:**
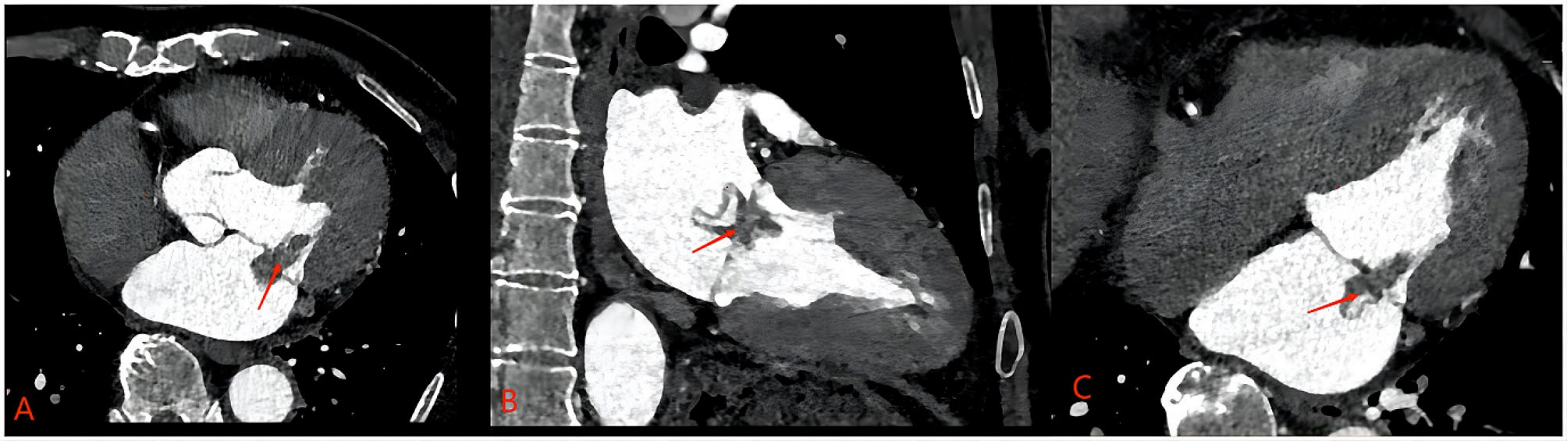
Computed tomography (CT) examination reveals an elliptical low-density filling defect in the mitral valve area with a cross-sectional size of about 2.9*1.2 cm (**A**,**B**,**C**) represented by red arrows

**Fig. 3:**
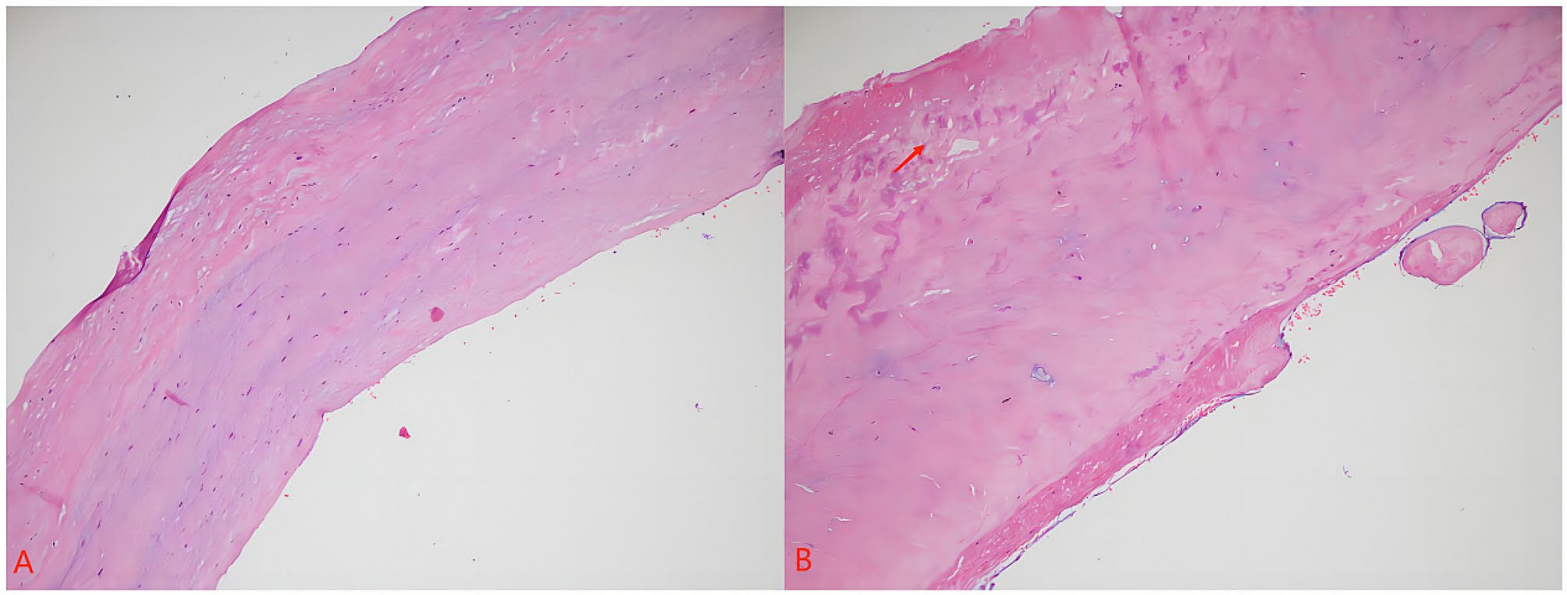
(**A**) HE staining shows the normal mitral valve structure in the resected part of the cyst wall. (**B**) The resected part of the cyst wall shows the smooth inner and outer wall. The tissue presents cyst like structure with no clear coated epithelium. There is fibrous hyaline degeneration and mucous degeneration of cystic wall and small foci of hemorrhage (red arrow)

A 66-year-old male patient presented with exertional chest tightness, shortness of breath, and right lower limb weakness for two weeks. He was diagnosed with a cardiac mass two months ago in another hospital. He had a history of hypertension from last 20 years which was under control with treatment. He underwent radical rectal surgery for rectal cancer five years ago and experienced intermittent fecal incontinence after surgery. On physical examination, his blood pressure was 124/67 mmHg, temperature 36.6 C^o^, pulse 64 bpm, RR 20/m. On percussion, the dullness of the left heart border was slightly extended indicating left heart enlargement. On auscultation, heart sounds were normal in all areas with normal rhythm and moist rale in both lung fields. Abdominal ultrasound revealed liver and left renal cyst. The prostate gland was mildly enlarged with calcified plaques. Coronary angiography (CAG) was performed without any remarkable findings. Transthoracic echocardiography (TTE) showed a mitral valve hypoechoic space occupying lesion with clear borders. Color doppler showed no blood flow signals around or inside the cystic mass. Contrast enhanced echo showed no contrast medium perfused into the cyst with moderate mitral regurgitation (MR) and ascending aortic dilation as shown in Fig. 1. Computed tomography (CT) examination revealed an elliptical low-density filling defect in the mitral valve area of the left atrium with a cross-sectional size of about 2.9*1.2 cm as shown in Fig. 2. Based on echocardiography, and CT report a preliminary diagnosis of cardiac cystic tumor was made. He underwent thoracoscopic surgery under general anesthesia and a cardiopulmonary bypass. The arterial and venous cannulation was done through the right femoral artery and vein respectively. A 4 cm incision was made in the 4th intercostal space (ICS) at the right anterior axillary line, a 3 cm incision in the 3rd ICS in the mid auxiliary line, and a 3 cm incision in the 2nd ICS in the midclavicular line. The aorta was cross-clamped and the cardiac arrest was achieved with the help of cardioplegia infused through the aortic root. There was no thrombus found in the left atrium. A cyst of about 20*20 mm at the P1 position of the posterior mitral leaflet was observed with mild enlargement of the mitral annulus. The mitral valve showed moderate regurgitation. The cyst was removed and the posterior leaflet of the valve was repaired with a complete semirigid 28 mm annuloplasty ring placed. The saline leakage test showed good competency of the mitral valve. The postoperative histopathology report showed a 2.5*2*0.7 cm gray and white cyst with smooth inner and outer wall. The tissue presented cyst like structure with no clear coated epithelium. There was fibrous hyaline degeneration and mucous degeneration of cystic wall and small foci of hemorrhage which confirmed the diagnosis of the mitral valve blood cyst (Fig. 3). The patient recovered well without any complications. The postoperative echocardiography showed no MR. Patient was doing well and was asymptomatic on six months follow up.

## Discussion

As a congenital malformation, cardiac blood cysts are common in infancy which regress with time but is very rare in children older than six months and in adults. They are small round reddish-brown nodules usually found on the atrial surface of the atrioventricular valves. They are usually sessile but can be pedunculated. In adults it is often solitary but multiple blood cysts have been reported which were present on both leaflets of the mitral valve [12]. Less than 100 cases of cardiac valve blood cyst have been reported in adults yet. Most of the cardiac blood cyst are found on atrioventricular valves [13], but it can be found in other parts of the heart such as aortic valve [14], pulmonary valve [8], left atrium [15], right atrium [16], right ventricle [17], papillary muscle [7], and atrial septum [9]. It is very rare entity and has not yet included in cardiac tumors classification by many articles [1, 18]. Even though it is rare but few hundred cases of cardiac blood cyst in the literature should be enough to include it in benign cardiac tumors list to increase awareness of the healthcare workers.

The cause of cardiac blood cyst is not very clear but there are multiple theories how they are formed. One very early concept by Luschka was that, it occurs as extravasation of blood from the vessels which they believed were present in the valve cusps [6]. Berti thought that, they may developed in the valves during vascular regression process [6]. Gross believed that, it was due to the obliteration of the valvular blood vessels [6]. Haushalter and Thiry’s theory was that, the blood was pressed by the heart chambers in the cervices and channels in the cusps that leads to blood cyst formation. They called it “Hematomes” [6]. As a known fact that the heart valves are avascular structure except from very short extension of the vessels in its bases. Dow and Harper believe that, the theories which explain the cardiac blood cysts are originated from the dilatation of previously present valvular blood vessels is unlikely [6]. They agree with the concept of Haushalter and Thiry. They believe during the embryonic stage, the traction of chordae tendineae make a furrow in the meshed fibrous structure and the fusion of the edges of the furrow lead to the development of cardiac blood cyst which was seen by serial sectioning of the valve cusps [6]. We believe this is probably the true mechanism because some cases of acquired blood cyst have been reported after surgery [19–21]. Inflammation, surgery etc. may cause the same furrows in the cusps of the valves which can lead to blood cyst formation. In older children and adults, it is possible that the congenital blood cyst which has been formed during embryonic stage get enlarge and result in clinical presentation but a part of these cases may be acquired due to other reasons.

Most cases are asymptomatic and the cyst is diagnosed incidentally when the investigation is performed for some other pathology. Cardiac tumors are generally diagnosed after symptomatic presentation of pulmonary or systemic embolization [1]. Patients can present with dyspnea, palpitation, syncope, stroke, hemiparesis, myocardial infarction, and valve obstruction or regurgitation. This patient presented with weakness of the right leg. Arteriovenous ultrasound did not show any thrombus of the leg vessels however brain CT showed lacunar cerebral infarction which may explain the condition. We believe the hemodynamic changes caused by the blood cyst leads to the formation of small thrombis and subsequently lacunar cerebral infarction.

It is very challenging to distinguish cardiac blood cyst form other cardiac tumors such as hemangioma, myxoma, vegetation, thrombus, cardiac varices, or hydatid cyst etc. [9]. Suspected cardiac tumors diagnosis should begin with investigation for exclusion of thrombus or vegetation [1]. Valve hemangiomas are occasionally found in the heart, and valve cysts are sometimes difficult to distinguish from valve hemangiomas however, valve cysts generally have no blood flow signals by echocardiography. Transthoracic echocardiography is the first line of investigation like for all other cardiac masses [1, 5]. Chang et al. believe that echocardiography is a convenient preoperative and follow up tool of investigation but it is not a very good choice for differential diagnosis [9]. Though, the final and definitive diagnosis can be made by histopathological examination, but due to the technical challenges of cardiac biopsy, preoperative diagnostic modalities such as CT and magnetic resonance imaging (MRI) is used for preliminary diagnosis [22–24]. It can also help in better understanding of the pathology and surgical decision making. Poterucha et al. believe that MRI is the most useful among the available modalities for cardiac tumors as it can help to demarcate the anatomy, and surgery is serving both diagnostic as well as therapeutic option [18].

As the cardiac tumors can embolize and cause complications, surgery is recommended especially for large cyst in symptomatic patients [1]. About 70% of cases of mitral valve blood cyst were managed surgically according to one research [13]. Some researchers believe that small asymptomatic blood cyst could be followed without surgical intervention. We believe that, because the natural history and hemodynamic effect of the blood cyst is not predictable, it is better to perform surgery to avoid future complication which would need surgery at that time. Most of the cases of mitral valve blood cyst have been operated through median sternotomy approach except for few which has been done with minimally invasive surgery [24, 25]. This case was performed through minimally invasive approach and is an addition to already available cases.

## Conclusion

Cardiac blood cyst is a very rare benign tumor in adults with less than 100 valvular cases reported so far. It should be considered in differential diagnosis when cystic cardiac tumor is encountered in a patient. Surgery is advised for large, symptomatic, and left side heart blood cyst to avoid complications.

## Data Availability

All data generated and analysed during this study are included in this published article.

## Abbreviations

TTE: Transthoracic echocardiography
CT: Computed tomography
MR: Mitral regurgitation
ICS: Intercostal space
MRI: Magnetic resonance imaging
